# Difference in F-Actin Depolymerization Induced by Toxin B from the *Clostridium difficile* Strain VPI 10463 and Toxin B from the Variant *Clostridium difficile* Serotype F Strain 1470

**DOI:** 10.3390/toxins5010106

**Published:** 2013-01-11

**Authors:** Martin May, Tianbang Wang, Micro Müller, Harald Genth

**Affiliations:** 1 Institute of Toxicology Hannover Medical School, Hannover D-30625, Germany; E-Mails: martinmay@gmx.de (M.M.); wangtb113@hotmail.com (T.W.); 2 Institute of Biophysical Chemistry, Hannover Medical School, Hannover D-30625, Germany; E-Mail: mueller.micro@mh-hannover.de

**Keywords:** Rho protein, mono-glucosylation, latrunculin B, *C. botulinum* C2 toxin, *C. limosum* exoenzyme C3

## Abstract

*Clostridium difficile *toxin A (TcdA) and toxin B (TcdB) are the causative agent of the *C. difficile*-associated diarrhea (CDAD) and its severe form, the pseudomembranous colitis (PMC). TcdB from the *C. difficile* strain VPI10463 mono-glucosylates (thereby inactivates) the small GTPases Rho, Rac, and Cdc42, while Toxin B from the variant *C. difficile* strain serotype F 1470 (TcdBF) specifically mono-glucosylates Rac but not Rho(A/B/C). TcdBF is related to lethal toxin from *C. sordellii* (TcsL) that glucosylates Rac1 but not Rho(A/B/C). In this study, the effects of Rho-inactivating toxins on the concentrations of cellular F-actin were investigated using the rhodamine-phalloidin-based F-actin ELISA. TcdB induces F-actin depolymerization comparable to the RhoA-inactivating exoenzyme C3 from *C. limosum * (C3-lim). In contrast, the Rac-glucosylating toxins TcdBF and TcsL did not cause F-actin depolymerization. These observations led to the conclusion that F-actin depolymerization depends on the toxin’s capability of glucosylating RhoA. Furthermore, the integrity of focal adhesions (FAs) was analyzed using paxillin and p21-activated kinase (PAK) as FA marker proteins. Paxillin dephosphorylation was observed upon treatment of cells with TcdB, TcdBF, or C3-lim. In conclusion, the Rho-inactivating toxins induce loss of cell shape by either F-actin depolymerization (upon RhoA inactivation) or the disassembly of FAs (upon Rac1 inactivation).

## 1. Introduction

*Clostridium difficile *toxin A (TcdA) and toxin B (TcdB) are the causative agent of the *C. difficile*-associated diarrhea (CDAD) and its severe form, the pseudo-membranous colitis (PMC) [1]. TcdA (MM 308 kDa) and TcdB (MM 270 kDa) are single chained proteins toxins with an AB toxin-like structure consisting of a *N*-terminally located glucosyltransferase domain and a *C*-terminal delivery domain [2]. The delivery domain harbors domains for receptor binding, a translocation domain, and a cysteine proteasome domain [3,4]. The delivery domain mediates cellular entry of the glucosyltransferase domain. TcdA and TcdB from the *C. difficile* strain VPI10463 mono-glucosylate the small GTPases Rho, Rac, and Cdc42, while Toxin B from the variant *C. difficile* serotype F strain 1470 (TcdBF) specifically glucosylates Rac but not Rho(A/B/C) [2,5]. Glucosylation at Thr-37 in RhoA and Thr-35 in Rac1 and Cdc42, a pivotal amino acid residue within the switch I domain, results in functional inactivation of each Rho protein, as it blocks interaction of the Rho protein with its effector and regulatory proteins [6,7]. *C. difficile* toxins with distinct Rho protein specificities are therefore often exploited as tools in cell biology research to analyze the involvement of Rho proteins in cellular processes [8,9,10,11].

Rho proteins are important regulators of actin nucleation and polymerization, with RhoA regulating the formation of stress fibers, Rac1 controlling formation of lamellipodia, and Cdc42 regulating filopodia formation [12]. Rho proteins further regulate cell-matrix adhesion, which involves initial cell-matrix binding and cell spreading. Integrins thereby cluster together in “focal complexes” (FCs) at the leading edge. These focal complexes grow into mature focal contacts, also called “focal adhesions” (FAs) [13]. In FAs, clustered integrins anchor actin filaments to the cell membrane and link them with the extracellular matrix (ECM) by adapter proteins such as talin and vinculin. The adapter protein paxillin links integrins to signaling proteins, forming a scaffold for Src kinases, the focal adhesion kinase (FAK), or the p21-activated kinase (PAK) [14,15,16]. Paxillin phosphorylation by PAK, FAK, or Src family kinases is important for paxillin localization and FA assembly [17]. In addition, paxillin and FAK locally regulate the activity of Rho proteins by FAK-induced phosphorylation and paxillin-mediated recruitment of Rho-regulating proteins, including GEF and GAP proteins [18,19]. 

Glucosylation of Rho proteins results in the loss of actin stress fibers, lamellipodia and filopodia, the disassembly of FAs, in reduced cell-matrix adhesion, and finally in rounding of cultured cells [2,20,21]. These morphological changes are regarded to involve F-actin depolymerization, as TcdB treatment results in an increased level of cellular G-actin [22]. 

In this study, the cellular levels of F-actin are analyzed upon F-actin staining by rhodamine-phalloidin using a fluorescence-based assay. For the first time, we here provide evidence on reduced F-actin levels in cells treated with the RhoA/Rac1/Cdc42-glucosylating TcdB. Most remarkably, the level of cellular F-actin is not reduced in cells treated with isomeric TcdBF, that glucosylates Rac, but not RhoA. These observations lead to the conclusion that the major pool of the cellular F-actin equilibrium is controlled by RhoA activity. 

## 2. Results

### 2.1. TcdB-Induced F-Actin Depolymerization

**Figure 1:**
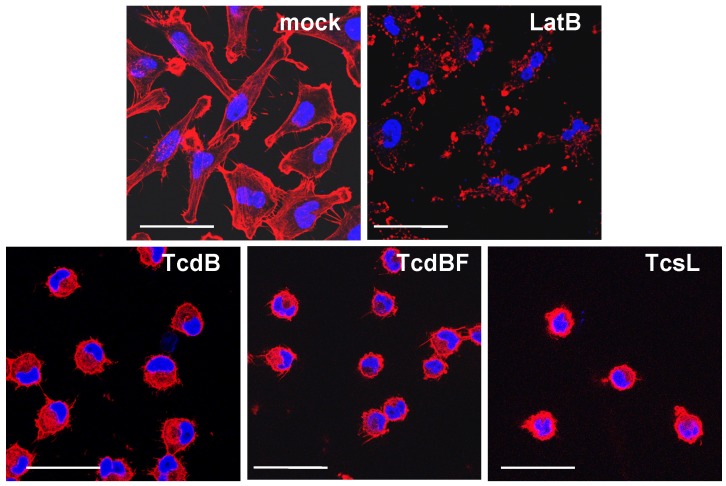
F-actin depolymerization induced by TcdB, TcdBF, TcsL, and Latrunculin B (LatB). HeLa cells were treated with the indicated toxins for 4 h. The actin cytoskeleton and the nuclei were visualized using fluorescence microscopy upon staining with rhodamine-phalloidin or DAPI, respectively. The size bar corresponds to 50 μm.

**Figure 2:**
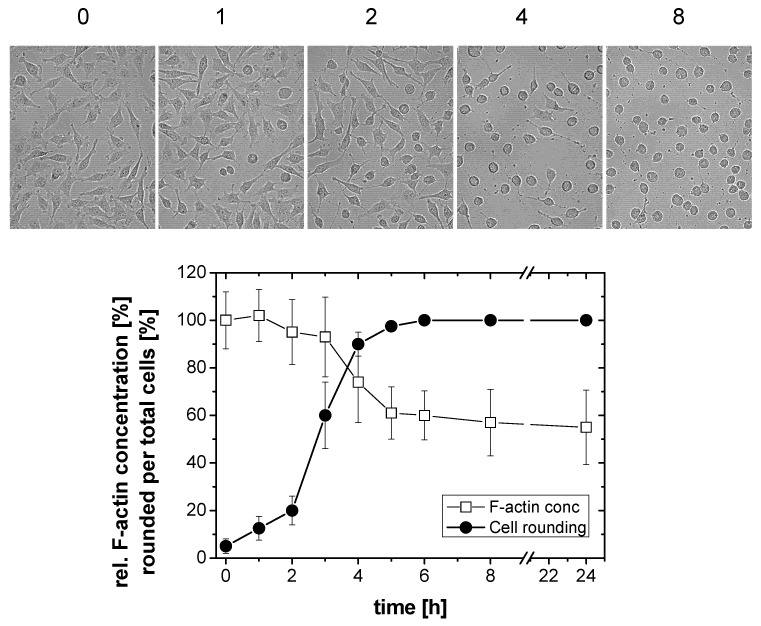
F-actin depolymerization induced by TcdB. HeLa cells were treated with TcdB (10 ng/mL) for the indicated time. Cell morphology was visualized using phase contrast microscopy. The cytopathic effect was quantified as the number of rounded per total cells. Filamentous F-actin concentration was measured by the rhodamine-phalloidin assay. The concentration of F-actin in non-treated cells was set 100. Values are the mean ± SD from three independent experiments made in triplicates.

Treatment of HeLa cells with TcdB resulted in a loss of actin stress fibers, lamellipodia and filopodia, and finally in cell rounding (Figure 1), as analyzed upon staining of F-actin with rhodamine-phalloidin and nuclei with DAPI. In TcdB-treated cells, F-actin was localized at the plasma membrane and in the cytosol (Figure 1). Morphological changes induced by TcdB was quantified in terms of the ratio of rounded per total cells, also referred to as cytopathic effect [2]. TcdB time-dependently induced cell rounding, with the complete cell population being rounded after TcdB treatment for 5 h (Figure 2). TcdB-induced rounding of HeLa cells was reflected by a reduced length of the longitudinal axis of cells (Figure S1). Untreated HeLa cells exhibited a length of the longitudinal axis of about 45 to 50 μm. Upon TcdB treatment, HeLa cells were rounded and exhibited a diameter of about 20 μm (Figure S1). The reduction in the length of the longitudinal axis of cell with spread morphology can thus be exploited as quantitative measure for the TcdB-induced cytopathic effect of spread cells. 

To determine whether TcdB-induced cell rounding was reflected by F-actin depolymerization, the cellular F-actin was stained by rhodamine-phalloidin and the F-actin concentration was determined by a fluorescence-based quantitative assay. This assay has widely been exploited to analyze the increase of cellular F-actin upon treatment of cells with growth factors [23] as well as the reduction of cellular F-actin induced by actin modifying toxins [24,25]. The cellular F-actin level time-dependently decreased in TcdB-treated HeLa cells (Figure 2). The F-actin level was thereby reduced to about 60% of the F-actin level of non-treated cells after 4 h of TcdB treatment (Figure 2). 

Prolonged incubation of TcdB-treated cells for up to 24 h did not result in a further decrease of the F-actin concentration (Figure 2). Upon prolonged TcdB treatment, some cells detached from the matrix. In terms of the applied method, cell detachment would result in seemingly decreasing F-actin concentrations, pretending F-actin depolymerization. To avoid such false positive results, detached cells were re-collected and added to the population of attached cells. 

**Figure 3:**
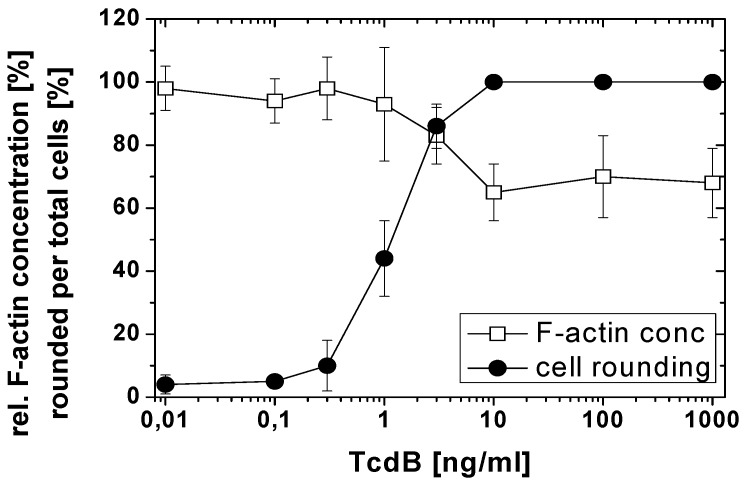
F-actin depolymerization induced by TcdB. HeLa cells were treated with the indicated concentrations of TcdB for 4 h. The cytopathic effect was quantified as the number of rounded per total cells. F-actin concentration was measured by the rhodamine-phalloidin assay. The concentration of F-actin in non-treated cells was set 100. Values are the mean ± SD from three independent experiments made in triplicates.

**Figure 4:**
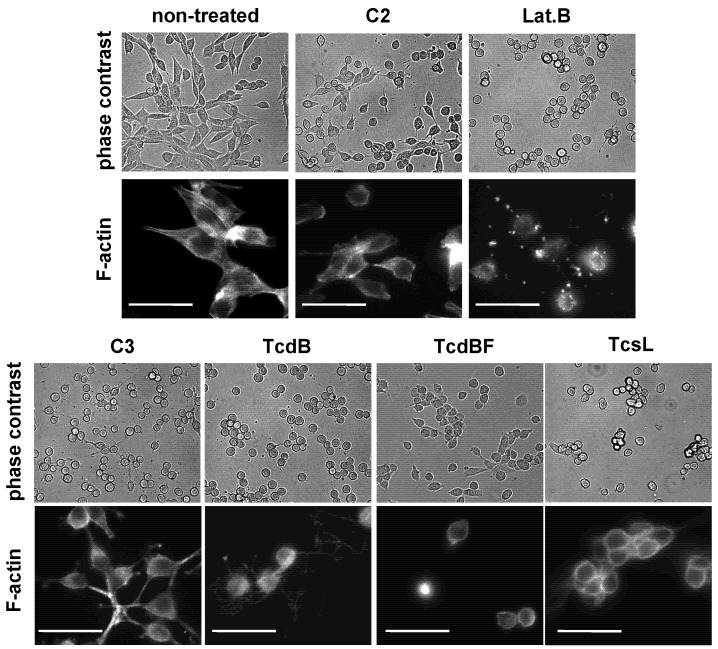
Toxin-induced changes of cell morphology. NIH3T3 fibroblasts were treated with the indicated toxins for 4 h. Cell morphology was visualized using phase contrast microscopy (upper panel) and fluorescence microscopy upon staining of F-actin with rhodamine-phalloidin (lower panel). The size bar corresponds to 50 μm.

**Figure 5:**
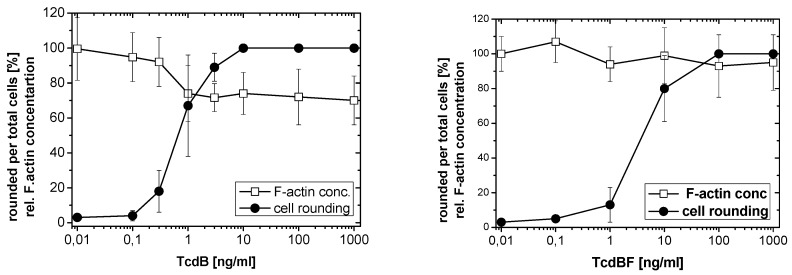
Difference in F-actin depolymerization induced by TcdB and TcdBF. Fibroblasts were treated with the indicated concentrations of TcdB (left hand graph) or TcdBF (right hand graph) for 4 h. The cytopathic effect was quantified as the number of rounded per total cells. F-actin concentration was measured by the rhodamine-phalloidin assay. The concentration of F-actin in non-treated cells was set 100. Values are the mean ± SD from three independent experiments made in triplicates.

TcdB-induced effects were further analyzed in a TcdB concentration-dependent manner after 4 h of toxin treatment (Figure 3). The F-actin concentration was reduced to about 60% of the F-actin concentration of non-treated cells at TcdB concentrations ≥10 ng/nL. TcdB-induced F-actin depolymerization was also observed in cell lines from different origins. TcdB concentration-dependently induced cell rounding and F-actin depolymerization in NIH3T3 fibroblasts (Figure 4 and Figure 5), in Hep2 cells (Figure S2), and in adherently growing rat basophilic leukemia (RBL) cells (Figure S3). TcdB-induced F-actin depolymerization is thus independent of the cell origin. 50% of cellular F-actin thus persists as rhodamine-phalloidin-stainable F-actin pool in TcdB-treated cells. 

**Figure 6:**
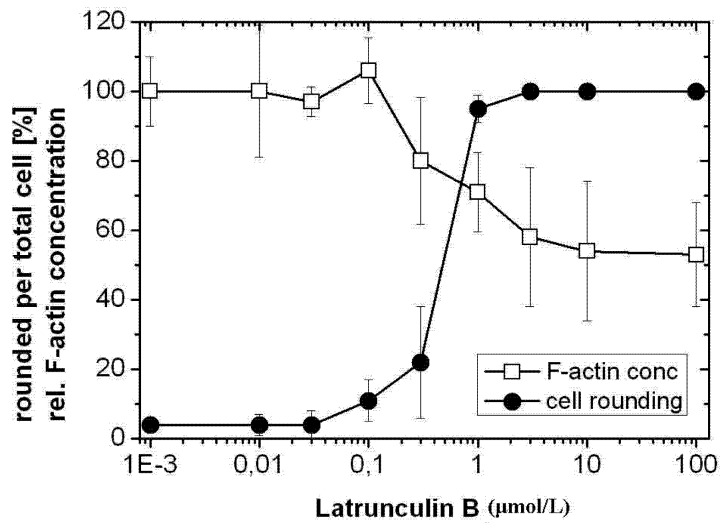
F-actin depolymerization induced by Latrunculin B. HeLa cells were treated with the indicated concentrations of Latrunculin B for 4 h. The cytopathic effect was quantified as the number of rounded per total cells. F-actin concentration was measured by the rhodamine-phalloidin assay. The concentration of F-actin in non-treated cells was set 100. Values are the mean ± SD from three independent experiments made in triplicates.

Latrunculin B (Lat B) is a cell-permenant macrolide that disrupts F-actin polymerization due to binding with monomeric G-actin. Furthermore, Latrunculin B concentration-dependently induced cell rounding and F-actin depolymerization in HeLa cells, with a reduction to about 50% of the initial F-actin concentration of non-treated cells in the presence of relatively high latrunculin B concentrations (Figure 6). Comparable to TcdB-treated cells (Figure 3 and Figure 5), a cellular F-actin pool of about 50% persisted as rhodamine-phalloidin-stainable F-actin pool also in cells treated with Latrunculin B. 

To further show that TcdB treatment resulted in F-actin de-polymerization, HeLa cells were lysed in F-actin stabilization buffer, followed by centrifugation to separate the F-actin (particular fraction) from the G-actin pool (soluble fraction). Actin signal were densitometrically quantified and given as the ratio of F-actin per G-actin. TcdB faintly reduced the F-actin level and increased the G-actin level in this assay, which resulted in moderate reduction of the ratio of F-actin per G-actin (Figure S4). Latrunculin B moderately reduced the level of F-actin and increased level of G-actin (Figure S4), which resulted in a decreased ratio of F-actin per G-actin (Figure S4). Taken together, different experimental settings were applied to evaluate the cytopathic effect of TcdB. The results showed that cell rounding induced by TcdB correlates with decreased F-actin concentrations.

### 2.2. No F-Actin Depolymerization in Cells Treated with the Variant *C. difficile* Toxin B and *C. sordellii* Lethal Toxin

Variant Toxin B from the *C. difficile* serotype F strain 1470 (TcdBF) differs from TcdB, as it glucosylates Rac1 but not Rho(A/B/C) [2,5]. Comparable to TcdB, TcdBF induced rounding of HeLa cells (Figure 1) and of NIH3T3 fibroblasts (Figure 4) with F-actin being localized at the plasma membrane and in the cytosol (Figure 1). HeLa cells (Figure S5) or fibroblasts (Figure 5) both were less sensitive to TcdBF than to TcdB, as the cell rounding—TcdBF concentration curves were shifted to higher TcdBF concentrations (Figure 5). This difference in the sensitivity of cells to TcdB and TcdBF is not matter of different glucosyltransferase activities but rather due to a different efficacies of receptor binding and/or internalization into the cell [26]. Remarkably, TcdBF-induced cell rounding was accompanied by reduced F-actin concentrations neither in fibroblasts (Figure 5) nor in HeLa cells (Figure S5). Consistently, the F-actin/G-actin ratio in TcdBF-treated HeLa cells was comparable to that of non-treated cells (Figure S4). TcdB and TcdBF thus differ in their capability of inducing F-actin depolymerization.

**Figure 7:**
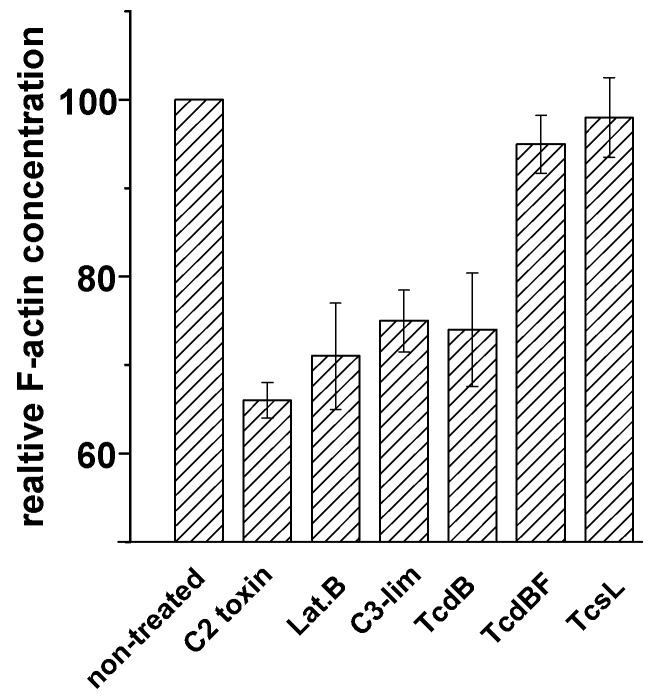
F-actin depolymerization induced by various toxins. Fibroblasts were treated with the indicated toxins for 4 h. Filamentous F-actin concentration was measured by the rhodamine-phalloidin assay. The concentration of F-actin in non-treated cells was set 100. Values are the mean ± SD from three independent experiments made in triplicates.

Lethal toxin from *C. sordellii* (TcsL) is closely related to TcdBF, as it glucosylates Rac1 and Ras proteins but not Rho(A/B/C) [27,28,29]. TcsL induced rounding of HeLa cells (Figure 1) and of NIH3T3 fibroblasts (Figure 4), with F-actin being present at the plasma membrane and in the cytosol. Comparable to TcdBF, TcsL-induced cell rounding was not accompanied by reduced F-actin concentrations (Figure 7). The Rac1-glucosylating toxins TcdBF and TcsL do not induce F-actin depolymerization. 

### 2.3. F-Actin Depolymerization Results in Paxillin Dephosphorylation

**Figure 8:**
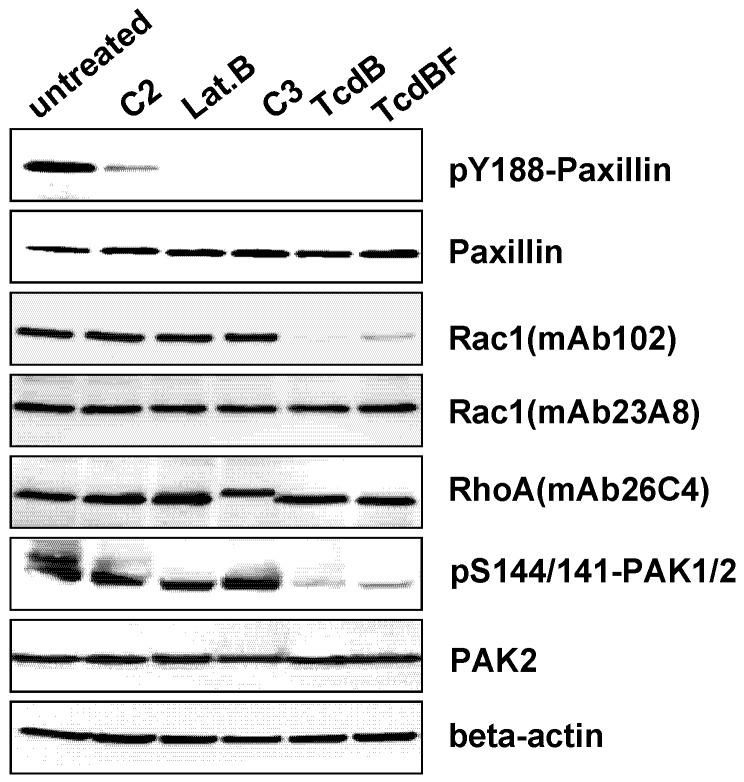
Toxin-induced paxillin dephosphorylation. Fibroblasts were treated with the indicated toxins for 4 h. Cellular levels of the indicated proteins were determined using Western blot analysis.

Besides the *C. difficile *toxins and latrunculin B, *C. botulinum* C2 toxin and C3-like ADP-ribosyltransferases affect the actin cytoskeleton. The actin ADP-ribosylating C2 toxin induced cell rounding (Figure 4) and reduced concentration of the cellular F-actin (Figure 7), consistent with published observations [24]. Upon treatment of fibroblasts with a cell permeable version of the Rho(A/B/C)-inactivating C3lim [30], fibroblasts were rounded and exhibited reduced F-actin concentration (Figure 7). Inactivation of Rho(A/B/C) thus appeared to be sufficient for the induction of F-actin depolymerization. C3lim-catalyzed ADP-ribosylation of RhoA was evidenced by reduced electrophoretic mobility of the RhoA band (Figure 8). F-actin depolymerization induced by TcdB might be attributed to the glucosylation of RhoA rather than of Rac1 or Cdc42. 

The observation that glucosylation of Rac1 by TcdBF did not result in F-actin depolymerization led to the question: how does the Rac1-inactivating TcdBF induce cell rounding? TcsL rapidly induces dephosphorylation of paxillin, a protein regulating focal adhesions (FAs), and leads to the disorganization of FAs [21]. Following the hypothesis that TcdBF comparably induced FA disassembly, the phosphorylation at Tyr-188 of the FA marker paxillin was exploited as a marker of FA assembly. A loss of pY188-paxillin was observed upon treatment with the Rac-inactivating TcdBF (Figure 8), showing that Rac1 inactivation was sufficient for paxillin dephosphorylation. Rac1 glucosylation was detected using the glucosylation-sensitive antibody Rac1-mAb(102). This antibody detects Rac1 glucosylation in terms of a loss of Rac1 detection (Figure 8) [31]. The loss of Rac1 detection upon TcdB treatment was due to glucosylation and not due to Rac1 degradation, as the cellular level of Rac1 was unchanged if the alternative Rac1-mAb(23A8) was applied (Figure 8). TcdB-catalyzed Rac1 glucosylation (*i.e.*, inactivation) was reflected by decreasing levels of pS144/141-PAK1/2, the active form of the Rac/Cdc42 effector protein PAK1/2 (Figure 8). 

A loss of pY188-paxillin was further observed in fibroblasts treated with TcdB, C3-lim, C2 toxin, and latrunculin B (Figure 8). These observations suggested that—besides Rac inactivation—inactivation of Rho(A/B/C) or actin de-polymerization each are sufficient for paxillin dephosphorylation. Comparable to TcsL, TcdBF-induced cell rounding appears to result from the disassembly of FAs. 

## 3. Discussion

Cell rounding is a robust morphological measure for the biological activity of the *C. difficile *toxins. It can be quantified either in terms of rounded per total cells [5] or in terms of the decreased cell diameter in any cultured cell lines with a spread phenotype including epithelial cells, fibroblasts, and adherently growing myeloid cells [32]. The cytopathic effect is a particularly useful measure, as its kinetics reflect the glucosylation of Rho proteins by clostridial toxins [5,29,33]. The quantification of the cytopathic effect by cell rounding moreover represents an established application using a simple and straightforward method with reliable results.

Decreasing F-actin concentration is also well established measures for the activity of bacterial toxins, in particular of actin modifying toxins [24]. The results of this study suggest that F-actin depolymerization can be further exploited as a biochemical measure for the activity of the Rho-glucosylating Toxin B isoforms (like TcdB) but not the Rac-glucosylating toxin B isoforms (like TcdBF) and TcsL. Actin depolymerization has been assumed as a hallmark of several clostridial toxins as cell rounding is accompanied by a loss of stress fibers, lamellipodia and filopodia. Importantly, the cytopathic effect of clostridial toxins is accompanied by a reduction of cell adherence. The reduction of cell adherence, might be the reason why another report suggests that the concentration of cellular F-actin strongly decreases in TcsL-treated cells, as analyzed by the phalloidin assay [21]. According to the Material & Methods section, Geny *et al.* does not recollect detached cells [21]. Under this condition, decreased F-actin concentrations observed in the phalloidin assay reflects cell loss due to cell detachment rather than F-actin depolymerization. In the modified method applied in this study, non-adherent cells are re-collected. Under this condition, the F-actin concentration in TcsL-treated cells is not reduced, thus comparable to the F-actin concentration in TcdBF-treated and non-treated cells. The recollection of detached cells thus avoids false positive results due to cell loss during experimental processing.

Based on the observation that inactivation of RhoA by C3-lim is sufficient for the induction of F-actin depolymerization, F-actin depolymerization induced by TcdB might be attributed to the glucosylation of RhoA rather than of Rac1 or Cdc42. Actin nucleation and actin polymerization are regulated by each RhoA, Rac1, and Cdc42 at distinct cellular sites through distinct cellular machineries [34]. The observation, that inhibition of RhoA by C3 or TcdB reduces the F-actin concentration, leads to the conclusion that the major pool of cellular F-actin is controlled by RhoA-dependent actin nucleation and polymerization in serum-cultivated cells. 

Our observation that treatment of cells with TcdBF and TcsL results in cell rounding suggests that RhoA inactivation-induced actin de-polymerization represents one but not the only trigger for toxin-induced cell rounding. Rac1 has been suggested to regulate FA assembly by different mechanisms, including PAK-dependent phosphorylation of paxillin and through the control of cellular phosphoinositide levels [18,19,21]. In particular, the Rac1 effector protein phosphatidylinositol 4-phosphate (PI4P) 5-kinase type I controls the generation of phosphatidylinositol 4,5-bisphosphate [21]. Rac1 glucosylation by TcsL has been reported to inhibit type I PI4P 5-kinase, which subsequently alters phosphoinositide metabolism leading to FA disassembly [21], a scenario that most likely also applies for TcdBF. In this study, TcdBF-induced FA disassembly is evidenced in terms of the dephosphorylation of the FA components PAK and paxillin. Paxillin dephosphorylation most likely results from Rac1 glucosylation, as paxillin is phosphorylated by several kinases including PAK [18,19]. Paxillin dephosphorylation most likely results from blocked PAK activity in TcdBF-treated cells. In turn, the critical role of Rac1 in sustaining the spread phenotype has recently been proven, as ectopic expression of non-glucosylable Rac1-G12V (not RhoA) preserves cells from TcdB-induced cell rounding [33]. Taken together, cell rounding induced by TcdBF or TcsL appears to be based on the loss of Rac-dependent cell-matrix adhesion and cell spreading (involving the disassembly of FAs and the loss of lamellipodia formation) but not on actin de-polymerization. 

Although cell rounding is induced by distinct mechanisms, TcdB-, TcdBF-, TcsL-, and C3lim-treated cells exhibit a comparable morphology with (remaining) F-actin being localized at the plasma membrane and in the cytosol. In the case of TcdB- and C3lim-treated cells, the loss of actin stress fibers is likely due to actin depolymerization. Stress fibers also disappear in TcdBF-/TcsL-treated cells, obviously without being depolymerized. The loss of stress fibers might be due to the disassembly of FAs, which anchor the stress fibers at the membrane. The yet unanchored actin filaments, that are not depolymerized, might localize to either the plasma membrane or into the cytosol.

## 4. Experimental Section

### 4.1. Materials

The following reagents were obtained from commercial sources: DAPI (40.6-diamidino-2-phenylindole) (Serva, Heidelberg, Germany); Cytochalasin D, Latrunculin B (Calbiochem, Nottingham, UK); rhodamine-conjugated phalloidin (Molecular Probes, Eugene, OR, USA). Antibodies: RhoA (mAb-26C4), Rac1 (mAb-23A8) (Santa Cruz Biotechnology, Santa Cruz, CA, USA); Rac1 (mAb-102) (BD Transduction Laboratories, Heidelberg, Germany); β-actin (mAb AC-40) (Sigma-Aldrich, Schnelldorf, Germany); Paxillin (2542), pY118-Paxillin (2541), PAK2 (2608) (New England Biolabs, Frankfurt, Germany); phospho-S144/141-PAK1/2 (ab40795) (Abcam, Cambridge, UK); horseradish peroxidise-conjugated secondary antibodies (Rockland Immunochemicals, Philadelphia, PA, USA). 

### 4.2. Toxin Purification

Toxin B from *Clostridium difficile* strain VPI 10463 (TcdB), variant toxin B (TcdBF) from *C. difficile* serotype F strain 1470, and lethal toxins from *C. sordellii* strain 6018 were purified as described previously [5,28]. In brief, a dialysis bag containing 900 mL of 0.9% NaCl in a total volume of 4 liters of brain heart infusion (Difco) was inoculated with 100 mL of an overnight culture of *C. difficile*, and the culture was grown under microaerophilic conditions at 37 °C for 72 h. Proteins were precipitated from the culture supernatant by ammonium sulfate at 70% saturation. The precipitates were dialyzed against Tris-HCl, pH 7.5, buffer overnight and loaded onto a MonoQ column (AP Biotech, New Jersey, NJ, USA). The toxins were eluted with 50 mM Tris-HCl, pH 7.5, buffer containing 500 mM NaCl and subsequently dialyzed against buffer (50 mM Tris-HCl [pH 7.5], 15 mM NaCl).

*Clostridium botulinum* C2 toxin, and cell-permeable *Clostridium limosum* exoenzyme C3 fusion toxin were expressed in *Escherichia coli* using the pGEX-2T vector system and purified with GSH−Sepharose beads (AP Biotech) as described [30].

### 4.3. Cell Culture

HeLa cells, NIH3T3 fibroblasts, and human hepatocarcinoma (HepG2) cells were cultivated in Dulbecco’s modified essential medium supplemented with 10% FCS, 100 μg/mL penicillin, 100 U/mL streptomycin and 1 mM sodium pyruvate. Rat basophilic leukemia (RBL-2H3) cells were grown as adherent monolayers on tissue culture flasks in minimum essential medium plus Earle’s salts (MEM plus Earle’s; Biochrom, Berlin, Germany) supplemented with 15% heat-inactivated fetal calf serum, 100 μg/mL penicillin, 100 units/mL streptomycin, and 1 mM sodium pyruvate. Cells were maintained in 5% CO2 at 37 °C. Upon confluence, cells were passaged. For all experiments, cells were seeded subconfluent. Phase contrast microscopy was performed on a Zeiss Axiovert 200 M.

### 4.4. Measurement of Filamentous Actin by Rhodamine-Phalloidin Fluorescence Assay

The F-actin concentration was determined after modifications of a rhodamine-phalloidin assay described previously [23,24]. Cells were seeded into 3.5 cm cell culture dished and treated with the toxin as indicated. Attached cell were suspended. Together with the detached cell, suspended cells were collected by centrifugation. The cells were washed with PBS and fixed with 3.7% paraformaldehyde in PBS for 10 min at RT. Cell were washed with PBS and incubated with 175 ng/mL rhodamine-labeled phalloidin (Molecular Probes) in PBS containing 0.5% bovine serum albumin (BSA) and 0.5 mg/mL saponin for 1 h at RT. Afterwards cells were washed with PBS containing 0.5 mg/mL saponin and bound rhodamine-labeled phalloidin was extracted with methanol over night at −20 °C. Fluorescence intensity of the samples was measured in the Fluorescence Reader Synergy 4 at 544 nm (excitation) and 590 nm (emission). Fluorescence intensity of toxin-treated cells was given relative to the fluorescence intensity of non-treated cells. 

### 4.5. Western Blot

Proteins were separated by SDS-PAGE and transferred onto nitrocellulose membranes. Membranes were blocked with 5% non fat dried milk for 60 min. Subsequently, the membrane was incubated with primary antibody at 4 °C over night and secondary antibody conjugated with horseradish peroxidase for 1 h at room temperature. Blots were analyzed by chemiluminescence reaction of ECL Femto (GE Healthcare, München, Germany) using Kodak Image Station 440 CF.

### 4.6. Immunocytochemistry and Immunofluorescence

HeLa cells were seeded onto cover slides and treated as indicated. Cells were fixed in 3.7% paraformaldehyde in PBS for 10 min at room temperature. The samples were washed with PBS and incubated with 5% BSA in PBS at room temperature for 1 h. Rhodamine-conjugated phalloidin and DAPI (1 μg/mL) were diluted in PBS and samples were incubated at room temperature for 1 h. Subsequently, cells were incubated for 1 h at room temperature. Samples were washed. Cover slides were fixed using Prolong Antifade (Invitrogen, Paisley, UK) and analyzed by fluorescence microscopy using Leica confocal microscope Inverted-2.

## 5. Conclusions

The observations of this study suggest that toxin-induced cell rounding is considered to result from either actin depolymerization (induced by covalent modifications of actin or RhoA) or the loss of cell spreading (induced by Rac1 glucosylation). Rounding of cultured cells induced by the *C. difficile* toxins is regarded to reflect the loss of epithelial barrier function of the colon upon infection with *C. difficile*. The virulence of *C. difficile* strains producing the standard toxin B (like TcdB) or variant Toxin B (like TcdBF) is comparable [35], showing that either molecular mechanism leading to cell rounding contributes to the virulence of CDAD. 

## Supplementary Files

Supplementary File 1 Supplementary Information (PDF, 186 KB)
