# Supplementary material for: Difference in F-Actin Depolymerization Induced by Toxin B from the Clostridium difficile Strain VPI 10463 and Toxin B from the Variant Clostridium difficile Serotype F Strain 1470
Source: Toxins (Basel). 2013 Jan 11;5(1):106–19. doi: 10.3390/toxins5010106 (PMC3564072; doi:10.3390/toxins5010106)
Supplement: Supplementary File 1 — Supplementary Information (PDF, 186 KB) [file toxins-05-00106-s001.pdf]

Supplementary Information

Figure S1. HeLa cells were treated with the indicated concentrations of TcdB for 4 h. The cytopathic effect was quantified as the number of rounded per total cells. Cell rounding was reflected by a reduced length of the longitudinal cell axis.

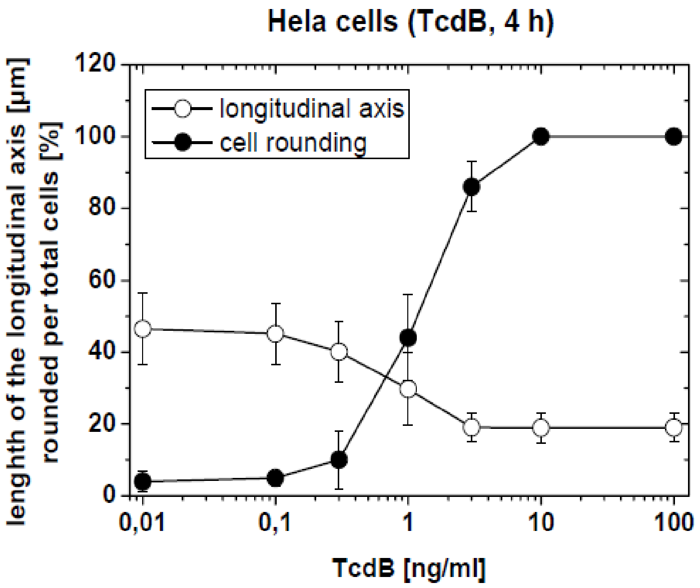

Figure S2. HEp-G2 cells were treated with the indicated concentrations of TcdB for 4 h. the cytopathic effect was quantified as the number of rounded per total cells. The F-actin concentration was measured by the rhodamine-phalloidin assay. The cellular level of F-actin in non-treated cells was set 100. Values are the mean ± SD from three independent experiments made in triplicates.

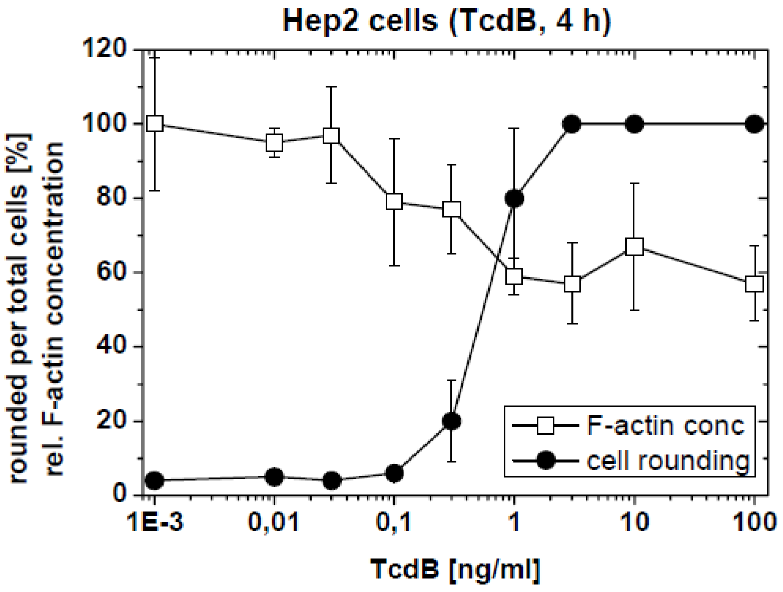

Figure S3. Rat basophilic leukemia (RBL) cells were treated with the indicated concentrations of TcdB for 4 h. The cytopathic effect was quantified as the number of rounded per total cells. The F-actin concentration was measured by the rhodamine-phalloidin assay. The cellular level of F-actin in non-treated cells was set 100. Values are the mean ± SD from three independent experiments made in triplicates.

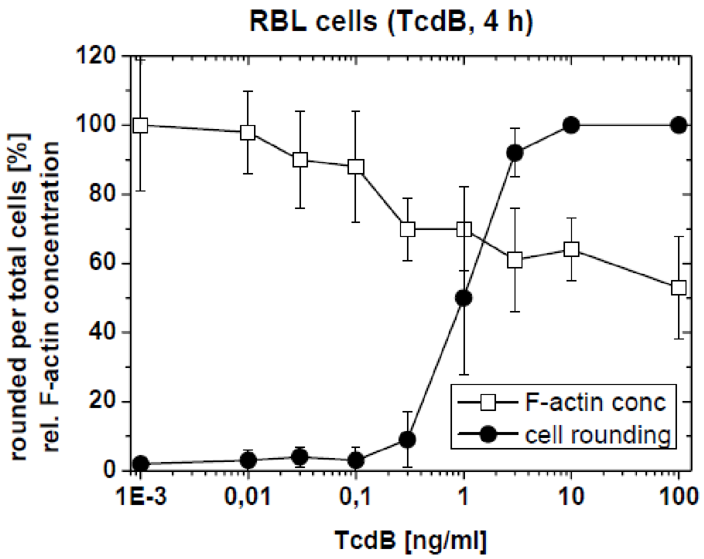

Figure S4. HeLa cells were treated with the indicated toxins for 4 h. Cells were lysed in F-actin stabilization buffer, followed by centrifugation to separate the F-actin (particular fraction P) from the G-actin pool (soluble fraction S). Actin signals were densitometrically quantified and given as the ratio of F-actin per G-actin. Values are the mean ± SD from three independent experiments.

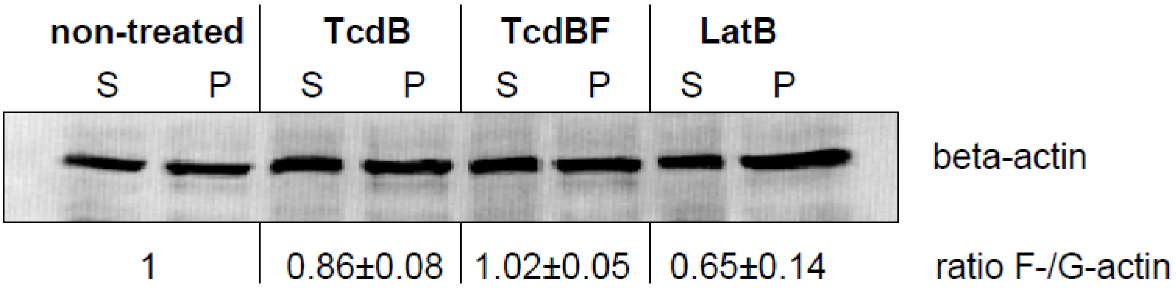

Figure S5. Hela cells were treated with the indicated concentrations of TcdBF for 4 h. The cytopathic effect was quantified as the number of rounded per total cells. The F-actin concentration was measured by the rhodamine-phalloidin assay. The level of F-actin in non-treated cells was set 100. Values are the mean ± SD from three independent experiments made in triplicates.

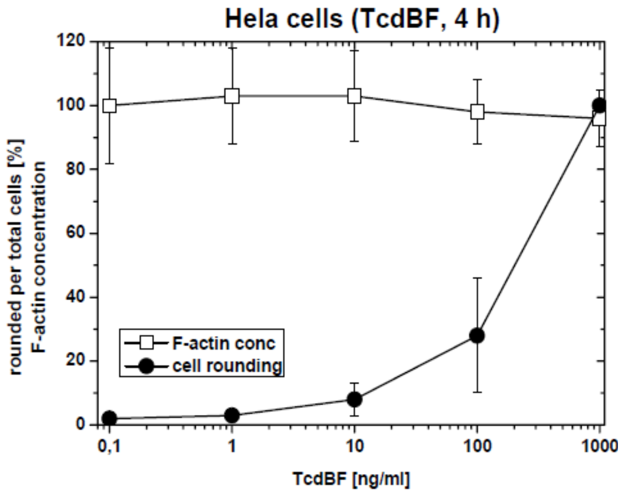

© 2013 by the authors; licensee MDPI, Basel, Switzerland. This article is an open access article distributed under the terms and conditions of the Creative Commons Attribution license (http://creativecommons.org/licenses/by/3.0/).
